# Adaptive Residual Correction Network for Efficient Single Image Super-Resolution

**DOI:** 10.3390/s26175451

**Published:** 2026-08-28

**Authors:** Jinsha Tian, Junyu Guo, Bingbin Feng, Hongjin Zhu, Yang Luo

**Affiliations:** 1 School of Data Science, Chengdu Technological University, Chengdu 611730, China; tjsha1@cdtu.edu.cn (J.T.); fengbingbin999@gmail.com (B.F.); zhjin2@cdtu.edu.cn (H.Z.); 2 School of Mechatronic Engineering, Southwest Petroleum University, Chengdu 610500, China; junyguo@163.com

**Keywords:** super-resolution, mapping error, residual correction, adaptive feature fusion

## Abstract

In recent years, vision transformers have demonstrated remarkable superiority to CNNs in single image super-resolution (SISR), yet their heavy computational and storage costs hinder practical deployment. In this work, we revisit CNN-based residual learning for SISR and identify a specific yet overlooked problem: the residual signal is often biased by mapping errors during model training, and this bias can propagate and accumulate through layers. To this end, we revisit the commonly used residual learning and feature fusion in SISR and propose an adaptive residual correction network (AdaRCN) in this work. First, we introduce a residual correction mechanism that adaptively compensates for the bias in the residual, which is utilized to ease error accumulation and improve mapping accuracy. On the other hand, we generalize the standard identity shortcut to a weighted channel concatenation followed by a 1 × 1 convolution, which is a more versatile strategy for adaptive feature fusion. Our AdaRCN is built entirely upon a naive CNN without complex architecture design and training strategies, thus ensuring efficient inference and parallelization. Extensive experiments verify the benefits and effectiveness of residual correction and adaptive feature fusion in improving the representational capability of our model, enabling it to achieve impressive performance comparable to advanced SISR models with moderate overhead.

## 1. Introduction

Single image super-resolution (SISR) is a classic and active topic in low-level computer vision, which aims to reconstruct a high-resolution (HR) image from a single low-resolution (LR) image. Although many solutions have been proposed for SISR tasks, it is currently still an active yet challenging hotspot due to the severe ill-posedness of the problem and its wide range of applications [1,2]. Beyond traditional imaging tasks, SISR and related image enhancement techniques also play a critical role in various engineering and industrial scenarios, such as fault diagnosis of high-speed train suspension systems [3], structural analysis of floating bridge modules [4], mooring tension prediction for offshore platforms [5], and low-illumination image enhancement for robust object detection [6]. Although deep learning has endowed the field with significant advancement in recent years, pursuing high-fidelity of SISR methods has still encountered bottlenecks because of the issue of information weakening as the network goes deeper. Therefore, it is of practical significance to improve the efficiency of network resources and the tradeoff between model performance and overhead, promoting the effort of developing lightweight models [7,8].

Some early methods typically adopted convolutional neural networks (CNNs) as the building architecture, such as SRCNN [9], CARN [10], DRRN [11], VDSR [12], etc. However, the nature of CNNs that extract local features and treat all intermediate features equally, limits the representational capability of these models. Residual learning [13], ref. [14] has significantly improved the performance of SISR models through increasing the depth of networks. For example, Kim et al. [12] used a global residual connection to build the first residual SISR model, which has a network depth of 20 layers. Moreover, RCAN [15] employed more residual connections and reached an inconceivable depth of over 400 layers. It also integrates channel attention [16] into the model for the first time to improve the utilization efficiency of model assets. Subsequently, various attention mechanism-based approaches have been proposed, such as spatial attention [17], pixel-level attention [18], hierarchical attention [19], etc. Nevertheless, these methods often involve complex operations that lead to slower inference, which is not conducive to the development of efficient lightweight models.

Recently, transformer [20] models have begun to prevail in the field of image super-resolution (SR) due to their superior capabilities to capture long-range or global dependencies [21,22,23]. However, as the critical component of transformers, multi-head self-attention (MHSA) generally keeps quadratic complexity, resulting in more computational and storage overhead than ordinary attention-based models. Although these models often have corresponding lite versions that can remedy the tradeoff between model overhead and performance, e.g., SwinIR-light [21], SRFormer-light [23] and Omni-SR [24], they still suffer from the impact of complex MHSA operations, making it hard to attain efficient inference and parallelism comparable to naive CNNs.

Both synergistic strategies, i.e., residual learning [13] to scale up deep models and attention mechanisms [16] to effectuate hierarchical features, can be considered as a recalibration on the backbone mapping, which makes the recorrected features more conducive to subsequent inference. The difference is that residual learning is a fine-grained (element-wise) and linear recalibration, while the attention mechanism normally behaves as a coarse-grained (channel-wise, spatial-wise, and layer-wise) but nonlinear one. Intuitively, fine-grained recalibration should be more suitable for low-level visual tasks with dense mapping. Due to the presence of extensive nonlinear features in images, such as spots, edges, corners, and spines, nonlinear recalibration also ought to be more preferred. Although residual learning and attention mechanisms are widely used in the field of image SR, few works inspect them from the perspective of feature recalibration.

**Figure 1:**
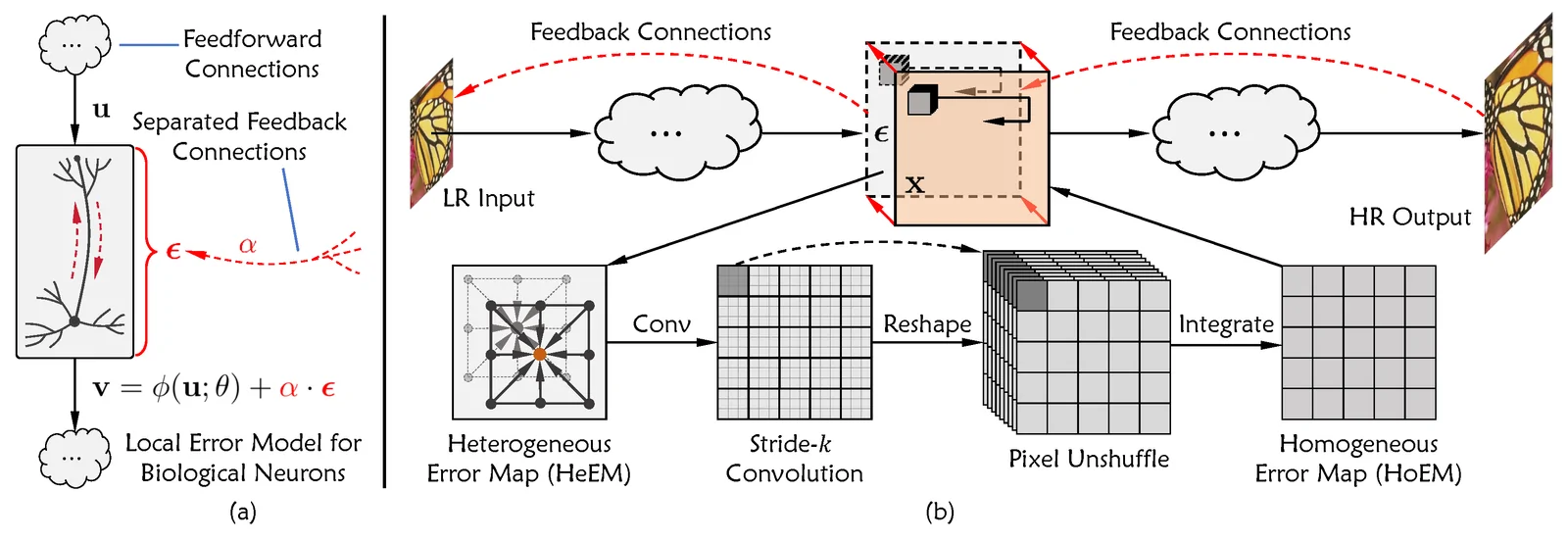
Schematic diagram for local computational error. (**a**) The perturbation synapse and the local mapping operate in different neural circuits. (**b**) The perturbation error (ϵ) is assumed to be implicitly contained in intermediate feature x. Due to the heterogeneity between ϵ and x, it is more reasonable that they have different behaviors during model inference. In the context of image SR, we adopt an implicit stride-*k* convolution for ϵ to discriminate the commonly used stride-1 convolution for x.

In neurobiology, there always exist some deviations during the local computation of a neural circuit, which are typically recorrected by *separate neural circuits* so that it can adapt to different tasks. This is called the Local Error Model (LEM) and is one of the main differences between biological neural networks and artificial neural networks, as shown in Figure 1. In this work, we design a fine-grained nonlinear recalibration to simulate LEM. It is a simple operation akin to pixel-unshuffle and converts intractable heterogeneous errors to homogeneous errors that can be directly removed from the features. Additionally, we extend residual learning with a structural component composed of a weighted channel concatenation (WCC) followed by a 1 × 1 convolutional layer. This serves as a fine-grained linear recalibration that cooperates with the nonlinear one above to improve the effectiveness of hierarchical features. Meanwhile, the two recalibrations are equipped with learnable coefficients, which are in favor of exploring more effective computation patterns for local mapping. Although the weighted interlayer connections are illustrated to impede back propagation in case of residual learning [14], we believe that they accord more with the manner that neurons behave in the human brain.

**Figure 2:**
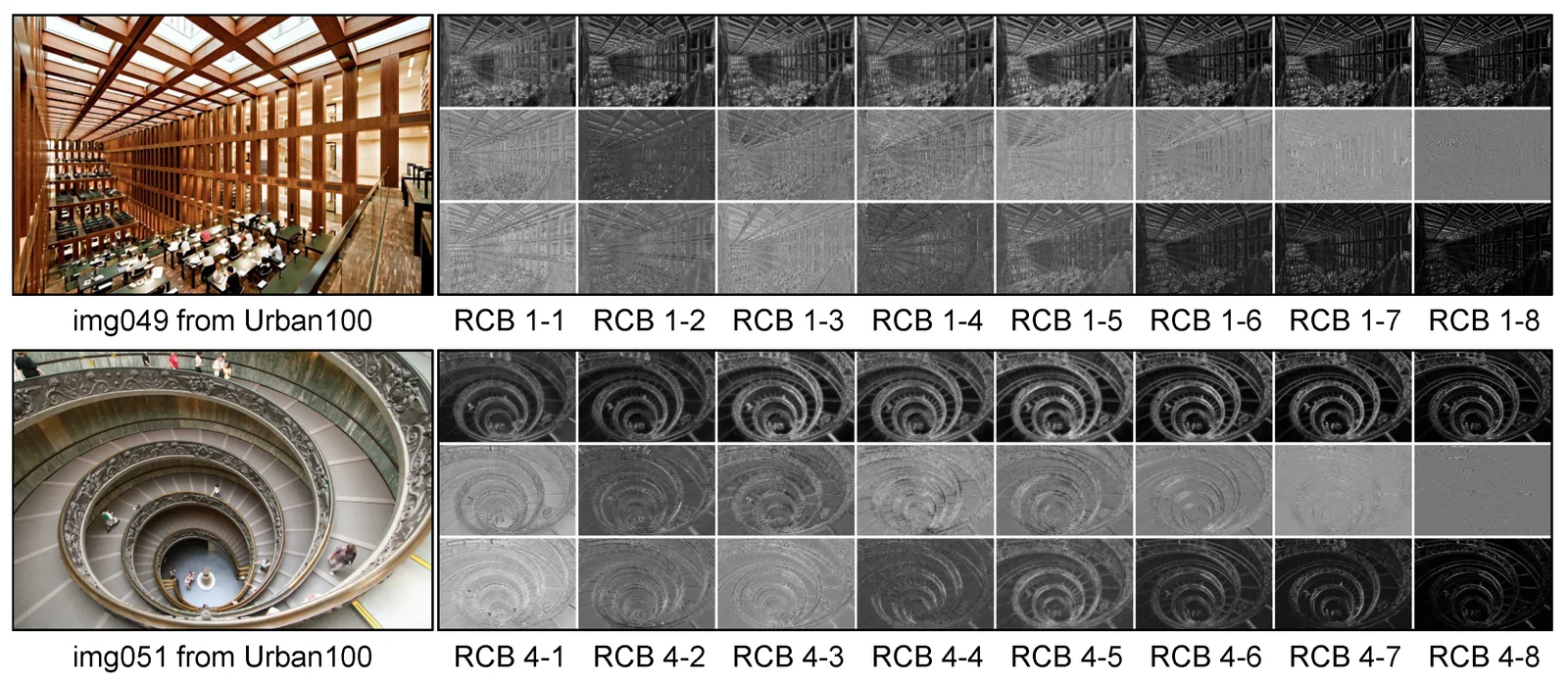
The visualization of residual correction for SR × 4. The features are displayed by averaging over all channels. The first and second numbers after “RCB” represent building group and block numbers, respectively. **Top**: feature residuals; **Middle**: residual errors; **Bottom**: corrected residuals. It can be seen that as the depth of the network increases, the errors of the residuals become smaller and the corrected residuals become closer to the original ones.

The property of traditional CNNs perceiving local receptive fields and sharing all convolution weights across entire feature maps endows them with favorable inductive bias, making them still dominant in the field of efficient SISR [25,26]. In this work, we still commit to fully exploiting the advantages of CNNs in respect to residual learning and feature fusion. Inspired by the intuition that there exist mapping errors in model inference of residual learning, we seek to correct these errors explicitly and adopt more versatile strategies to promote adaptive feature fusion. As shown in Figure 2, the corrected residuals increasingly resemble the original ones, implying more accurate inference for SISR tasks. The primary contributions of this work can be summarized as follows:

- We identify a critical yet overlooked issue in residual learning for SISR (i.e., the residual signal is biased by mapping errors during training) and propose a residual correction mechanism to compensate for this bias.
- We generalize the ordinary identity shortcut to a weighted channel concatenation followed by 1 × 1 convolutional layer, enabling adaptive feature fusion with learnable combination strengths across the entire network.
- A lightweight AdaRCN is presented for efficient SISR, which achieves competitive performance against state-of-the-art models based on a transformer with moderate computational overhead.

## 2. Related Work

Deep learning has revolutionized SISR over the past decade, with research progressing from deeper architectures to more efficient feature representations. This section reviews the related work from three key aspects that are most relevant to our approach, including SISR methods based on deep learning, interlayer connections in deep models, and feature recalibration for vision tasks.

### 2.1. SISR with Deep Learning

The pioneering contribution that uses deep learning [27] to deal with SR tasks is SRCNN [9], which is a three-layer CNN network that maps LR images to HR images in an end-to-end manner. Initially, the further improvement based on SRCNN focuses on increasing network depth [12] and sharing network weights [11,28], both of which can promote SR performance but enlarge model scale in different aspects. Even so, these methods also suffer from another obstacle that hinders further model upscaling, i.e., nonlinear inference in HR image space that inevitably increases computational burden [15,29]. This problem can be solved by performing upsampling at the end of the network, thus embedding nonlinear inference in LR image space, e.g., fractionally-strided (or transposed) convolution [30] and ESPCNN [29]. Benefitting from this, some deep models can improve model performance by significantly augmenting their network depth and parameters, such as EDSR [31], RDN [32], D-DBPN [33], RCAN [15], etc. However, the performance gain of these methods depends largely on the enlargement in model scale. For example, EDSR [31], NLSA [34], and OISR-RK3 [35] have more than 40 M model parameters, greatly increasing the model scale. The performance of SR models has currently reached a certain bottleneck. A more promising approach might be to combine image priors [36] or other theoretical tools [37].

It is difficult to further upgrade the scale of SR models, as information weakening and vanishing gradients make model training more and more problematic, even though the network is sophisticatedly built (e.g., residual learning, feature fusion, progressive training, etc.). Hence, the current mainstream concentrates on promoting the effectiveness of network assets or feature representation. The attention mechanism [16] is such a typical and commonly used technique that adjusts the feature responses towards the most informative and important components of the inputs. Zhang et al. [15] first incorporated the channel attention of SENet [16] into SR tasks and achieved excellent performance with only ∼16M parameters. Subsequent work attempted to extend attention to spatial dimensions [38] and even layers [19]. Moreover, nonlocal attention [34,39,40] is also exploited for image SR as it is capable of capturing long-range dependencies and executing more complicated and challenging attention. Behjati et al. [41] proposed a directional variance attention (DiVA) to extract cross-channel and spatial information simultaneously. Cai et al. [42] integrated the attention mechanism into a multi-branch model, aimed at perceptually pleasant SR recovery. Although these attention-based models can improve the effectiveness of feature representation similar to other large-scale models, the attention mechanism itself is derived from high-level visual tasks and recalibrates feature maps in a coarse-grained way. Additionally, some complex implementations for the attention mechanism also notoriously cause large amounts of computational burden and decrease the efficiency of model assets, e.g., nonlocal attention [34,39,40]. Recently, SISR methods based on deep learning have also been extended to other modalities and dimensions. For instance, Wang et al. [43] explored a 3D surface SR method via normal-based multimodal restoration, demonstrating the potential of cross-modal information for fine-grained texture recovery.

### 2.2. Interlayer Connections

A simple and direct manner to improve the performance of deep models is to increase model scale. However, as mentioned above, more problems will arise as model scale increases and more tricks are typically needed to guarantee effective training [44]. To ease the training difficulty, interlayer connections are widely employed in network design. Two representatives are residual connection [13,14] and channel concatenation [45]. Although residual connections are a preferred option in image SR, e.g., [11,12,15,31,46], prior works have shown that residual learning will cause representative redundancy and reduce the efficiency of model assets [47,48]. It indicates that the performance of residual SR models can be potentially improved [10,49] if model scale is relatively fixed. Channel concatenation is another kind of interlayer connection that concatenates features from different layers along the channel dimension, such as SRDenseNet [48], MemNet [46], MSRN [44], RDN [32], etc.

Theoretically, interlayer connections facilitate the effective fusion between linear and nonlinear features, shallow and deep features, short and long-range features, and thus improve the model performance. Nevertheless, most of the above methods ignore the role of adaptive connection strength that may be in more accord with the behavior of neurons in the brain. In this work, we also adopt interlayer connections to perform feature recalibration, but in an adaptive way. As can be observed from Section 3, the interlayer connections with learnable coefficients are also motivated from the model of local computational errors in neurobiology. Another role of these coefficients is to explore the combination pattern of network topology, promoting the representative capability of the model.

### 2.3. Feature Recalibration

Feature recalibration is designed to regulate the responses of local mappings of the network according to the interdependencies between hierarchical features so that feature representation is more effective and suitable for subsequent model inference. Emblematic examples include the attention mechanism [16] and lateral inhibition mechanism (LIM) [50], which are essentially inspired by the behavior of neurons in the brain.

Attention-based recalibration is initially proposed for high-level visual tasks, such as localization [51], segmentation [52,53,54], classification [16,55,56], etc. In most cases, it is coarse-grained due to the channel-wise, spatial-wise, or layer-wise operations. While some recent works [57,58] have proposed fine-grained attention mechanisms based on element-wise products, their performance improvement is very limited, and the adaptability of interlayer connections is not considered. Similarly, the LIM [50] realizes fine-grained and nonlinear recalibrations, while the Hartline–Ratliff Equation (HRE) [59] it tries to simulate is more complicated in computation, which goes against efficient execution. Residual learning [13] can also be viewed as a special case that performs fine-grained but linear recalibrations, where the current feature is directly recalibrated by preceding features via element-wise summation. Our implementation of feature recalibration is also inspired by the neurobehavior in the brain, but sublimated from the perspective of error compensation.

## 3. Adaptive Residual Correction Network

### 3.1. Motivation

Recent research shows that the learning process of the brain can implement the core principles underlying backpropagation [60], but in a significantly different manner. The main idea is that the brain possesses feedback connections separated from feedforward connections. And these feedback connections can induce neuron activities whose *locally* computed differences encode backpropagation-like error signals so that effective synaptic updates can be computed [61,62], as shown in Figure 1a. Formally, it can be expressed as follows:

(1) v=ϕ(u;θ)+α·ϵ,

where ϕ(·) denotes a local mapping in the network, and u and v represent the input and output of ϕ(·), respectively. θ is a weight for simulating synaptic feedforward connections. ϵ is the error signal induced by separated feedback synapses and α implies a regulator that simulates the strength of feedback connections. In the absence of backpropagation, neural circuits can calculate synaptic updates, e.g., in the following way:

(2) $$\underset{\theta }{\arg\min}\epsilon =\underset{\theta }{\arg\min}\frac{1}{\alpha }E\left[||v-{\phi \left(u;\theta \right)||}_{2}^{2}\right],$$

where E[·] indicates the expectation. Equation (2) indicates that the externally induced error allows the model to finetune synapses locally. Equation (1) is equivalent to the residual model [13] if α=1.0 and ϵ=u. However, this is less likely to happen in neurobiology since ϵ and u originate from different neural circuits. In traditional CNNs without separated feedback connections, depicting ϵ according to the observable u becomes an important issue.

Upon the above idea, our hypothesis is that *the intermediate feature* x *implicitly contains the local mapping error **ϵ**, which can be estimated from* x *and used to recalibrate the inference of the network*. Since x and ϵ come from different neural circuits, they should be neurobiologically *heterologous*. This indicates that each element in ϵ interferes discriminatorily with different neurons in the output, as shown in the heterogeneous error map (HeEM) of Figure 1b. For a k×k convolution, we have the following equation:

(3) y=(x−α·ϵ)∗w=x∗w−α·ϵ∗w,

where x and $y\in R^{H\times W}$ (the channel dimension is ignored for simplicity). The first and second terms should have different behaviors: x∗w is typically a stride-1 convolution for dense sampling, while ϵ∗w should be a stride-*k* convolution to avoid the same element in ϵ causing the same interference to different elements in y. To keep the same dimensions as x and y, ϵ should have a shape of kH×kW, as shown in the stride-*k* convolution in Figure 1b. However, ϵ is *implicitly* included in x and we cannot conduct stride-*k* convolution to learn a homogeneous error map (HoEM) directly. Inspired by the idea of ESPCNN [29], it can be approximated by learning a temporary error map with size of $H\times W\times k^{2}$ that serves as a trainable heuristic approximation to the conceptual heterogeneous error map $\epsilon \in R^{kH\times kW}$. Afterwards, we can learn a HoEM through a 1×1 conv, which could be directly used to calibrate x with x−α·ϵ, as shown in Figure 1b. Please note that this implementation is an approximation motivated by the conceptual analogy in Figure 4, rather than an exact spatial rearrangement operation, as the ground-truth error map is not directly available in practice.

We would like to clarify that the biological discussion merely serves as conceptual motivation rather than a claim of mechanistic equivalence. Our primary contribution is the computational framework and conceptual analogy, which are derived and validated within the context of image SR.

### 3.2. Overall Structure

**Figure 3:**
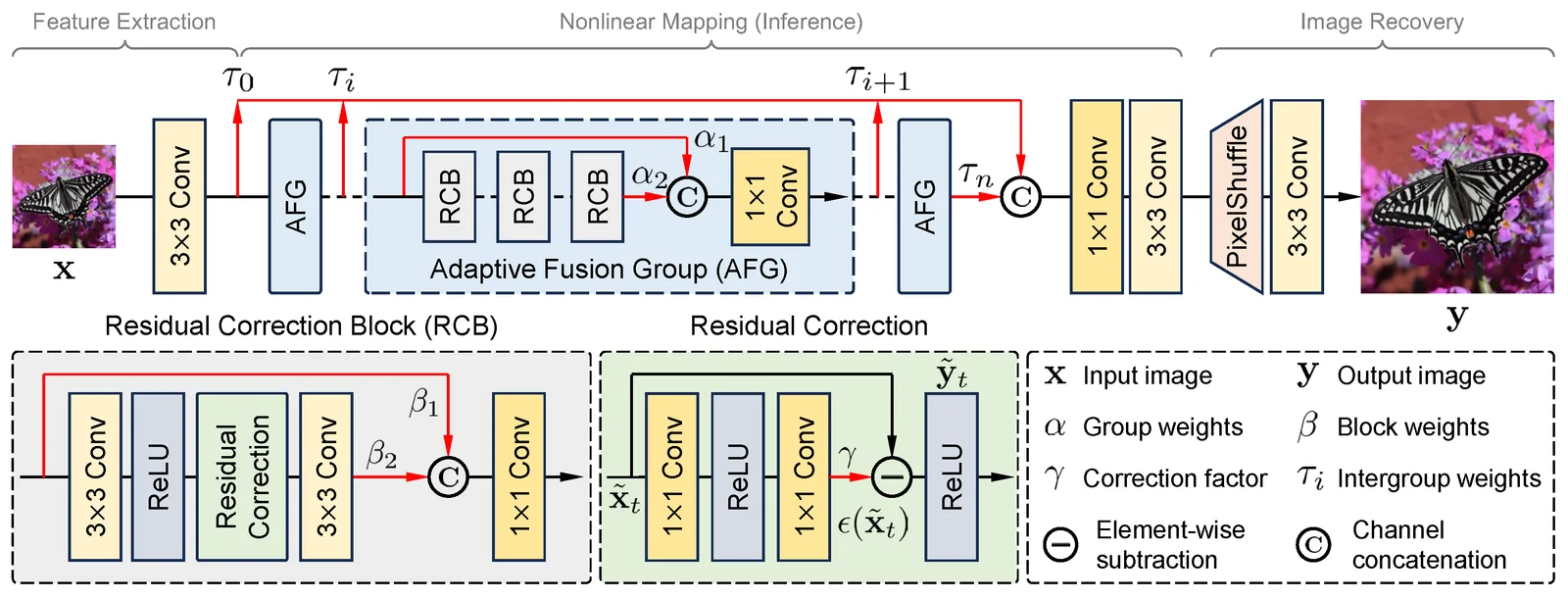
The overall architecture of the proposed AdaRCN and its building components. Red arrows indicate learnable weights for adaptive feature fusion and *n* is the number of AFGs. Meanwhile, we set *m* RCBs in each AFG, which are followed by a channel concatenation and a 1 × 1 convolution.

The overall structure of our AdaRCN follows the general paradigm of SISR models and consists of three stages: feature extraction, nonlinear inference, and image recovery, as shown in Figure 3. Given an LR image $x\in R^{h\times w\times 3}$, we first extract shallow features $x_{0}\in R^{h\times w\times c}$ with a 3 × 3 conv layer, where *c* denotes the number of feature channels. Subsequently, $x_{0}$ is processed by a series of adaptive fusion groups (AFGs), which produces a list of intermediate features $x_{1},x_{2},\ldots ,x_{n}$, where *n* is the total number of AFGs. Formally, the inference can be formulated as follows:

(4) $$x_{i}={\mathrm{AFG}}_{i}\left(x_{i-1}\right),1\leq i\leq n,$$

where ${\mathrm{AFG}}_{i}$(·) denotes the mapping function of the *i*-th AFG. The intermediate features are then adaptively integrated in the following manner:

(5) $$x_{d}=F_{d}\left(\mathrm{Concat}\left(\tau_{0}\cdot x_{0},\tau_{1}\cdot x_{1},\ldots ,\tau_{n}\cdot x_{n}\right)\right),$$

where $\tau_{i}\;\left(0\leq i\leq n\right)$ are used as learnable intergroup weights and $F_{d}$(·) corresponds to a 1 × 1 conv layer followed by a 3 × 3 conv layer. Concat(·) indicates the operation of concatenating features along the channel dimension; therefore, it produces a feature tensor with (n+1)×c channels. Finally, $x_{d}$ is fed into a PixelShuffle layer followed by a 3 × 3 conv layer to restore the HR target $y\in R^{H\times W\times c}$, where H=r·h,W=r·w, and *r* stands for the scaling factor. In each AFG, we deploy *m* stacked residual correction blocks (RCBs), which function as the basic module of our model. As illustrated in Figure 3, our RCB is built on the residual block of EDSR [31], but with a residual correction module, which endows our model favorable SISR inference.

### 3.3. Residual Correction

**Figure 4:**
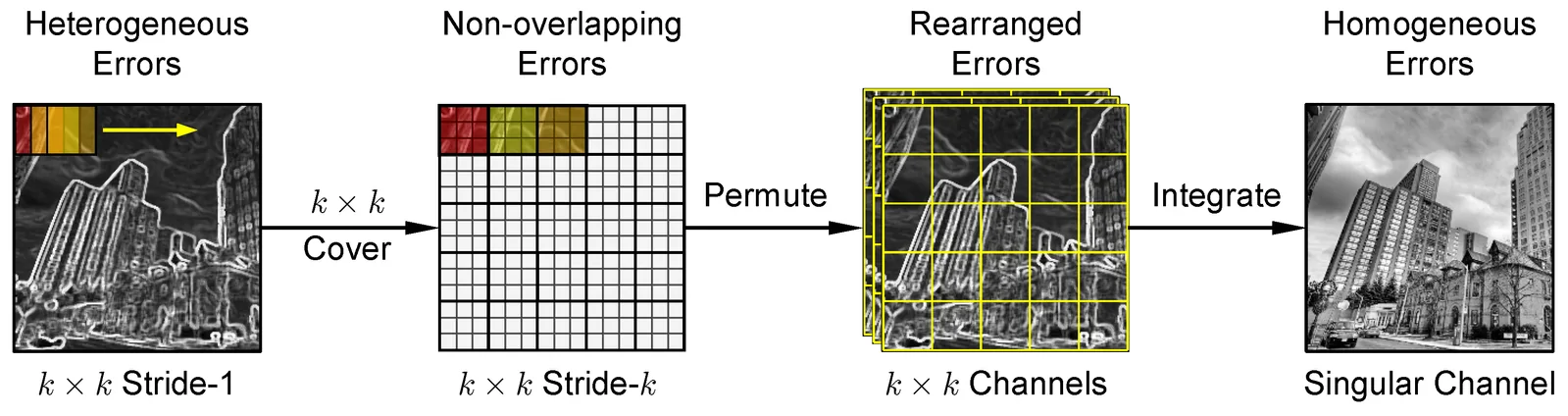
Diagram of mapping errors in nonlinear inference and their simulation computation. It is reasonable to consider the errors as heterogeneous and non-additive, so if a h×w×1 residual is convolved with a kernel of k×k, the latent errors can be organized as kh×kw×1, and further $h\times w\times k^{2}$. The process can be simply analogous to the inverse of PixelShuffle [29].

If a h×w×c residual feature is convolved with a k×k kernel, the errors within a k×k cover should be heterogeneous from those within other covers. If the stride of the convolution is 1 (which is the case in most SISR models), these heterogeneous features can be reorganized into a shape of kh×kw×c, and then rearranged into $h\times w\times c\cdot k^{2}$, as shown in Figure 4. The entire process is akin to the inverse of PixelShuffle [29], but related to the kernel size of the convolutions in RCBs. To simulate the process, we design a simple unit for residual correction. Given a residual feature ${\tilde{x}}_{t}\in R^{h\times w\times c}$, we use two 1 × 1 conv layers to adjust its channels to $c\mapsto k^{2}\cdot c\mapsto c$, and produce a homogeneous feature that can be directly subtracted by the original residual feature. The process can be formulated as follows:

(6) $${\tilde{y}}_{t}={\tilde{x}}_{t}-\gamma \cdot \epsilon \left({\tilde{x}}_{t}\right),$$

where ϵ(·) corresponds to the two 1 × 1 conv layers, and γ is a learnable parameter for adaptive residual correction. As exhibited in Figure 3, to impose non-negative and sparse constraints on the errors, we apply a ReLU function at the end of the unit.

### 3.4. Adaptive Feature Fusion

To fully mine the promotion of feature fusion on the performance of SISR models, we expand residual connections into a more generalized strategy referred as weighted channel concatenation, and apply this strategy across various levels of the network. Apart from Equation (5), we deploy it in both AFGs and RCBs:

(7) $$\begin{array}{cc} {\tilde{y}}_{t} & =\mathrm{PConv}(\mathrm{Concat}\left(\alpha_{1}{\tilde{x}}_{t},\alpha_{2}F_{\mathrm{AFG}}\left({\tilde{x}}_{t}\right)\right)), \end{array}$$

(8) $$\begin{array}{cc} {\tilde{y}}_{t} & =\mathrm{PConv}(\mathrm{Concat}\left(\beta_{1}{\tilde{x}}_{t},\beta_{2}F_{\mathrm{RCB}}\left({\tilde{x}}_{t}\right)\right)), \end{array}$$

where PConv(·) represents a point-wise convolution, and $F_{\mathrm{AFG}}\left(\cdot \right)$ and $F_{\mathrm{RCB}}\left(\cdot \right)$ stand for the nonlinear branches in AFGs and RCBs, respectively. $\alpha_{k}$ and $\beta_{k}$ (*k* = 1, 2) are learnable weights for adaptive feature fusion. Equations (7) and (8) can be summarized as ${\tilde{y}}_{t}=\mathrm{PConv}\left(\mathrm{Concat}\left(w_{1}{\tilde{x}}_{t},w_{2}H\left({\tilde{x}}_{t}\right)\right)\right)$, where H(·) stands for $F_{\mathrm{AFG}}\left(\cdot \right)$ or $F_{\mathrm{RCB}}\left(\cdot \right)$ and $w_{k}$ means $\alpha_{k}$ or $\beta_{k}$. Let us rewrite ${\tilde{x}}_{t}=\left[{\tilde{x}}_{t}^{1},\ldots ,{\tilde{x}}_{t}^{i},\ldots ,{\tilde{x}}_{t}^{c}\right]$ and $H\left({\tilde{x}}_{t}\right)=\left[{\tilde{x}}_{h}^{1},\ldots ,{\tilde{x}}_{h}^{j},\ldots ,{\tilde{x}}_{h}^{c}\right]$, both of which have *c* feature channels with spatial size of h×w. Denote the kernel of the 1 × 1 convolutional layer as $K\in R^{2c\times c}$ with 2c input channels and *c* output channels (in implementation, the shape of K is $\left[k,k,c_{\mathrm{in}},c_{\mathrm{out}}\right]$, where *k* is kernel size, and $c_{\mathrm{in}}$ and $c_{\mathrm{out}}$ denote the number of input and output channels. Since K is 1 × 1 here, we remove these singular dimensions for simplification). Omitting the biases, ${\tilde{y}}_{t}$ is obtained by the following:

(9) $${\tilde{y}}_{t}^{u}=\sum_{i=1}^{c}w_{1}\cdot {\tilde{x}}_{t}^{i}\cdot K\left(i,u\right)+\sum_{j=1}^{c}w_{2}\cdot {\tilde{x}}_{h}^{j}\cdot K\left(c+j,u\right),$$

where ${\tilde{y}}_{t}=\left[{\tilde{y}}_{t}^{1},\ldots ,{\tilde{y}}_{t}^{u},\ldots ,{\tilde{y}}_{t}^{c}\right]$ and ${\tilde{y}}_{t}^{u}$ stands for the *u*-th 2D feature map of ${\tilde{y}}_{t}$. Here, *u* is the index of the output channel. It is easy to see that $w_{k}$ mainly controls the influence of the locally computed error on model inference. The proposed fusion strategy can simulate various potential network structures presented in previous work. For instance, if adaptive recalibration is deleted, i.e., α=0: when $w_{1}=w_{2}=1.0$ and K in Equation (9) satisfies the following:

(10) $$K\left(i,j\right)=\left\{\begin{array}{cc} 1, & i=j\;\mathrm{or}\;i=j+c,\\ 0, & \mathrm{otherwise}, \end{array}\right.$$

where i=1,…,2c, j=1,…,c, then our fusion strategy degrades to the residual block of EDSR [31]. Meanwhile, if $w_{1}$ and $w_{2}$ act as learnable reweighting factors, then it degrades to the residual block in AWSRN [49]. The potential architectures indicate that our adaptive feature fusion can implicitly realize the combination of various typical structures, thereby improving the representation capability and learning efficiency of the model.

## 4. Experimental Results

### 4.1. Datasets and Metrics

Like most previous works, we employ 800 training images from DIV2K [63] as our training set. The training images are augmented by randomly horizontal and vertical flips, and $90^{\circ }$ rotations. Moreover, we also implement another kind of data augmentation, which is termed as data range complementarity. It is achieved by subtracting the original input image from the entire data range (e.g., 255.0 for 8-bit unsigned integers). Five benchmark datasets, i.e., Set5 [64], Set14 [65], BSD100 [66], Urban100 [67], Manga109 [68], are used for evaluation. The reconstructed results are evaluated with PSNR and SSIM [69] on Y channel of YCbCr space. To evaluate the computational overhead of SISR models, MultiAdds [10] is also introduced as an evaluation metric, where the size of HR images are assumed to be 720p, i.e., 1080 × 720 in spatial size.

### 4.2. Implementation Details

We implement our model by setting *m* = 8, *n* = 4. The last conv layer has three filters as it outputs RGB images. Feature channels are set to 32 elsewhere; therefore, the first pointwise conv in RCBs outputs $3^{2}\times 32=288$ channels. The sizes of all conv kernels follow the annotations of Figure 3, and zero-padding is applied to keep the spatial size of features unchanged. All learnable weights (including α, β, γ, and τ) are scalars and are initialized to 1.0, which corresponds to regular models without weighting coefficients. We randomly crop LR patches with size of 64×64 for model training, and batch size is set to 16. The loss is minimized by the Adam optimizer [70] with $\beta_{1}=0.9,\;\beta_{2}=0.999,\;\epsilon =10^{-8}$. We train our model with $10^{6}$ iterations, and the learning rate is initialized as $2\times 10^{-4}$ and halved at every $2\times 10^{5}$ training steps. The proposed models are implemented with PyTorch 2.3 and trained with CUDA 11.8 on a workstation equipped with a NVIDIA RTX A5000 GPU. The code of our AdaRCN is available at https://github.com/jinsha-tech/AdaRCN.git.

### 4.3. Comparison with Advanced Methods

We extensively compare our AdaRCN with existing efficient SISR methods, including CNN-based methods like LAPAR-A [7], TSAN [71], DiVANet [41], LatticeNet [8], HPUN-L [72], FMEN [73], OSFFNet [74], CoMoNet-S [75], SRConvNet-L [26], etc., and transformer-based models such as SwinIR [21], LBNet [76], ESRT [77], NGswin [78], CFIN [79], DIIN [80], and GMN [81]. These compared methods cover representatives of efficient SISR methods in recent years.

**Figure 5:**
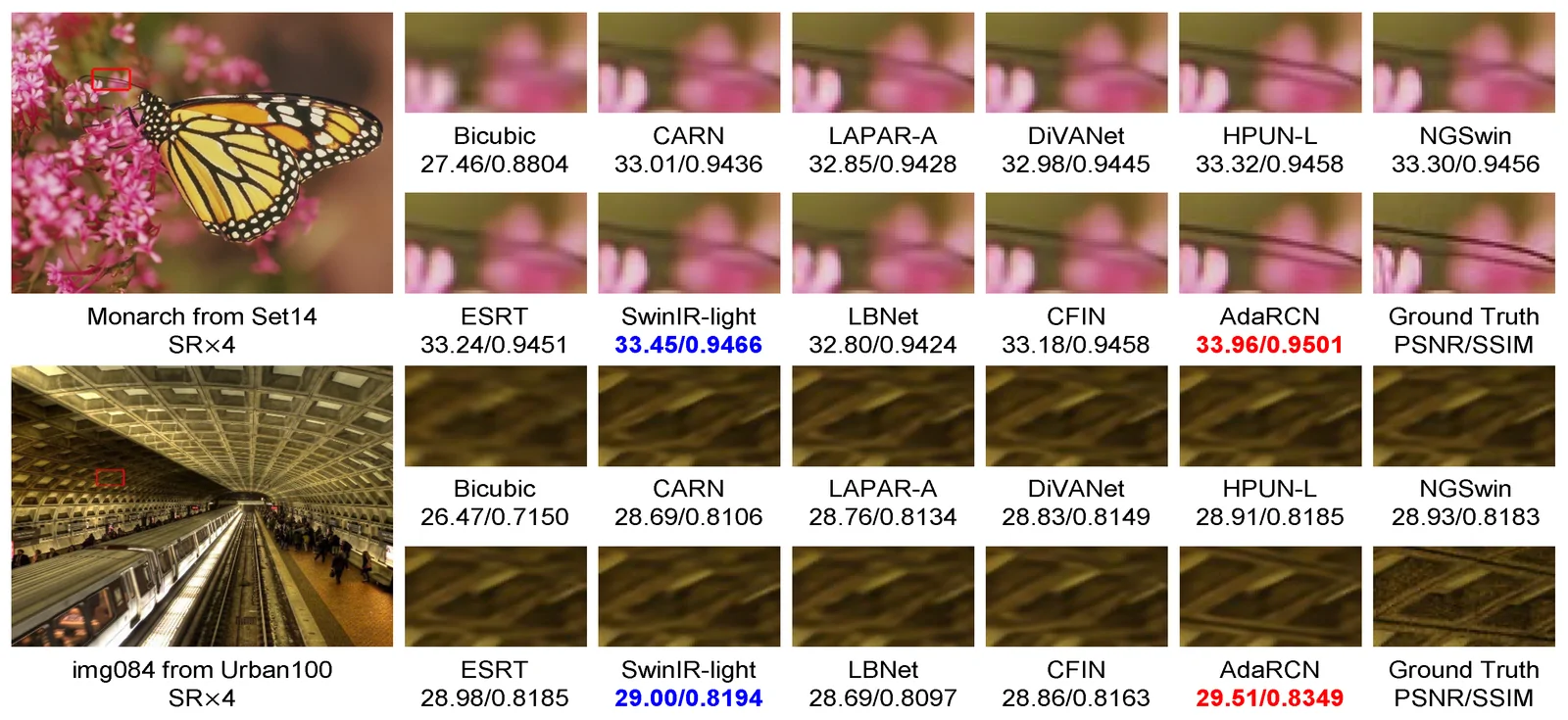
Visual comparisons between advanced lightweight SISR methods and our AdaRCN on “Monarch” from Set14 [65] and “img084” from Urban100 [67] with SR × 4. The best and second best results are marked in red and blue, respectively. Zoom in for best view.

**Figure 6:**
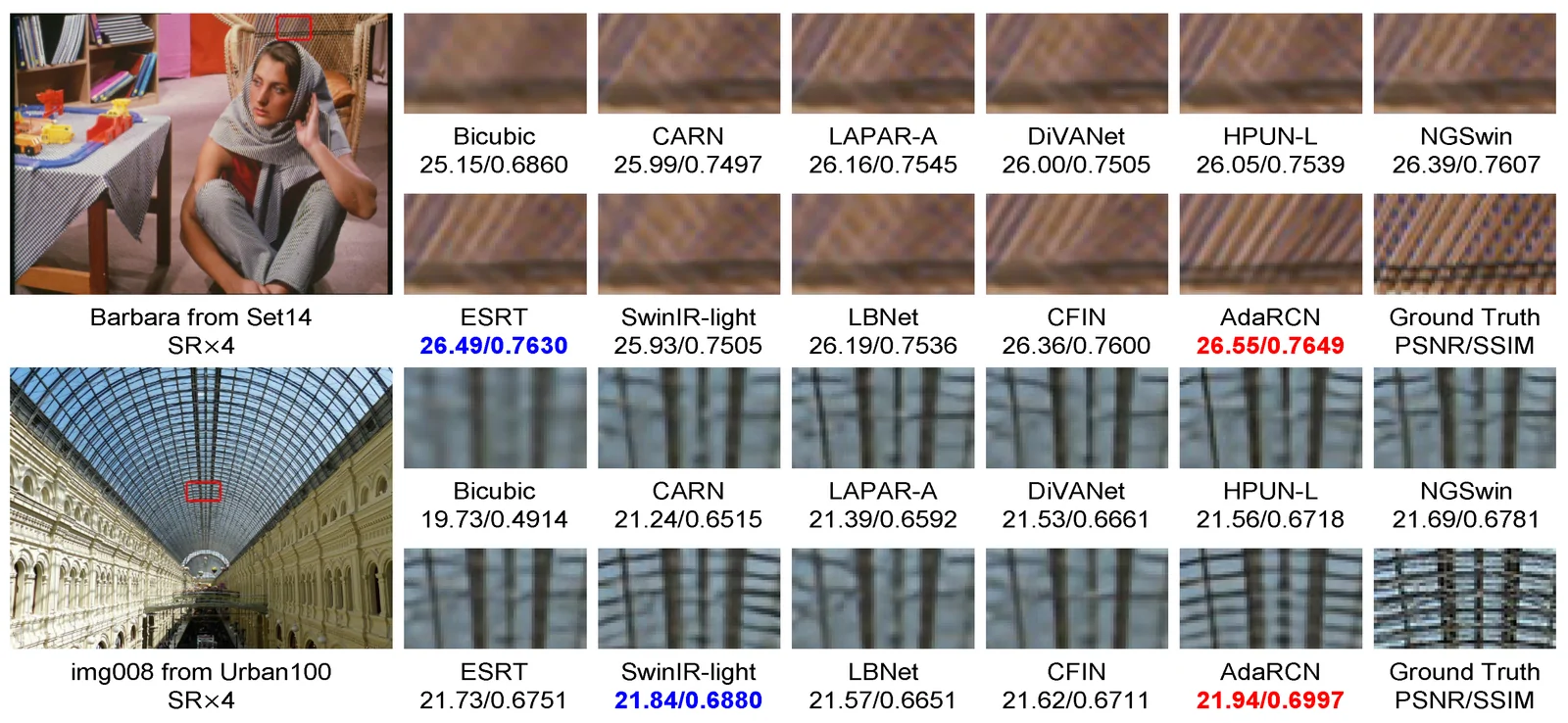
Visual comparisons between advanced lightweight SISR methods and our AdaRCN on “Barbara” from Set14 [65] and “img008” from Urban100 [67] with SR × 4. The best and second best results are marked in red and blue, respectively. Zoom in for best view.

**Quantitative Results:** Table 1 presents the quantitative results of the compared methods on five benchmark datasets, grouped according to CNNs and transformers. As can be observed, the performance of most CNN-based methods is relatively inferior. Only two methods, TSAN [71] and SRConvNet-L [26], exhibit performance close to our AdaRCN. However, the parameters and computational cost of TSAN [71] are approximately three times that of our AdaRCN. However, SRConvNet-L [26], despite having a lower computation cost than AdaRCN, demonstrates poorer performance and, to some extent, its network still draws inspiration from transformers. In most cases, our AdaRCN also exceeds advanced transformer-based methods. For instance, it significantly performs better than GMN [81], which has a similar parameter scale and computational cost. Despite having fewer network parameters, the inference latency of models based on the transformer architecture is usually greater than that of CNN-based models.
**Visual Comparison:** Figure 5 and Figure 6 show the visual results of our AdaRCN and compared models on test images from Set14 [65] and Urban100 [67] with SR × 4. Both objective metrics (PSNR and SSIM) and subjective visual quality are reported across four representative test images to prove the superiority of our method.

As shown in Figure 5, our AdaRCN demonstrates a clear visual advantage in recovering fine structural details on the “Monarch” from Set14 [65]. Specifically, the two antennae of the butterfly reconstructed by the proposed AdaRCN are remarkably sharper and more coherent, closely matching the Ground Truth, whereas other methods produce blurred or distorted contours. This visual superiority is well supported by the quantitative results: AdaRCN achieves the highest PSNR/SSIM of 33.96/0.9501, substantially outperforming the second-best SwinIR (33.45/0.9466). For the “img084” from Urban100 [67], which contains complex repetitive structures of a subway station skylight, most competing methods fail to preserve the correct periodicity and directional patterns, resulting in noticeable texture artifacts and misaligned grids. In contrast, our AdaRCN reconstructs the skylight structure with much higher fidelity and clearer edge definition, yielding the best quantitative scores of 29.51/0.8349, with a significant gap over the second-best SwinIR (29.00/0.8194).

In Figure 6, on the “Barbara” image that features intricate striped textures on the scarf and headband, our AdaRCN effectively restores the directional consistency and fine-grained patterns, while other methods tend to produce over-smoothed or wavy distortions. For the “img008” from Urban100 [67], which involves challenging building facades with repetitive windows and structural lines, our method recovers clearer boundaries and more regular patterns, whereas most competitors introduce blurring or ghosting effects. The quantitative results for the two images are also presented below the small patches and well support the results of visual comparison.

**Figure 7:**
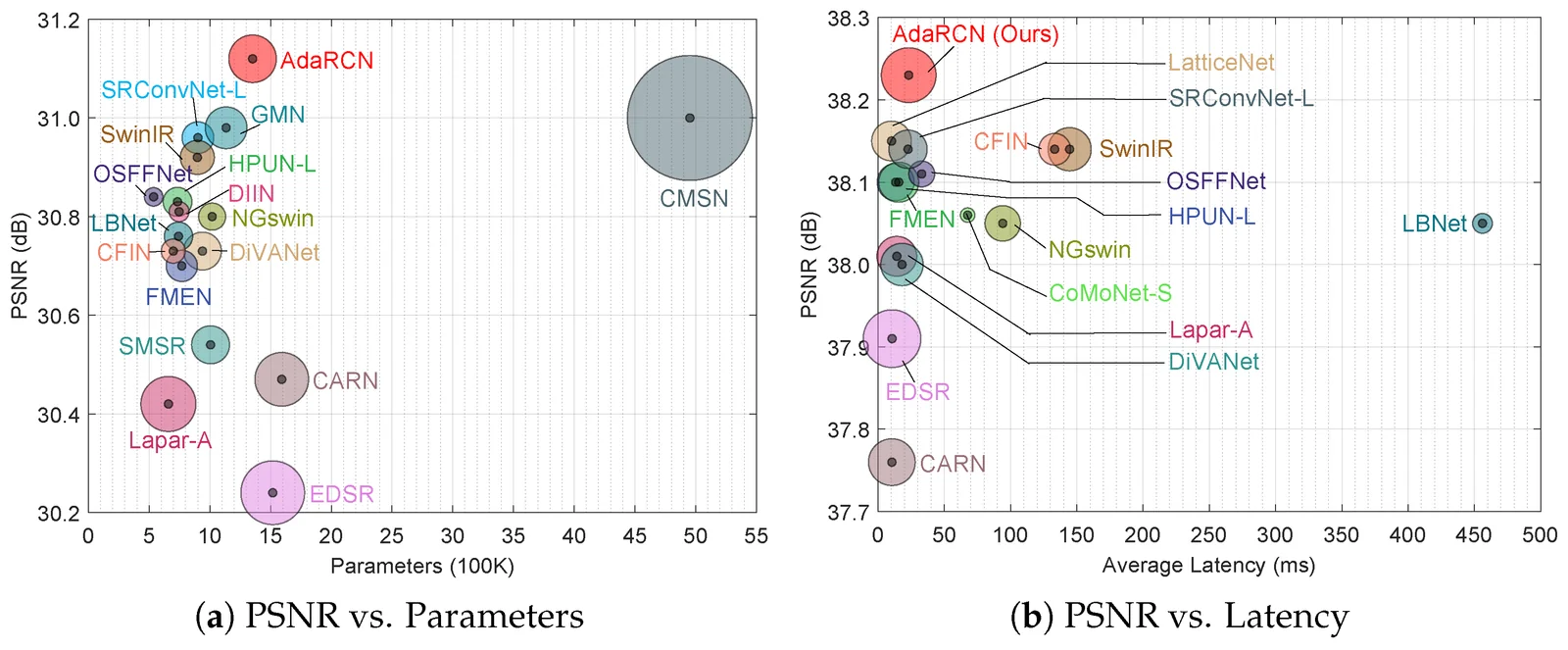
The tradeoff comparison between typical SISR models and our AdaRCN. (**a**) The tradeoff between model performance and overhead on Manga109 [68] with SR × 4. The radii of these circles hint the MultiAdds of the models; (**b**) the compromise between model performance and inference latencies on Set5 [64] with SR × 2 (averaged over 10 trials).

**Tradeoff Comparison:** Figure 7 demonstrates the tradeoff between model performance and overhead (including parameters and MultiAdds) on Manga109 [68] with SR × 4. It can be observed that our AdaRCN achieves the best performance by a substantial margin, with moderate overhead. Although CMSN [82] achieves a comparable PSNR of 30.10 dB, it requires about 4.95M parameters, which is more than three times the parameter and FLOPs budget of AdaRCN. Moreover, it holds the same comparison to GMN [81], which is the best performing approach among transformer-based models. Our AdaRCN has a similar number of model parameters and computational cost as GMN [81], but the PSNR is 0.14 dB higher. This indicates that our AdaRCN can also be comparable to advanced architectures based on transformers in terms of the balance between model performance and overhead.

On the other hand, we also present the balance between model performance and inference latency in Figure 7b, where the latencies are computed as the average time per image in Set5 [64] over 10 independent trials (SR × 2). It can be seen that our AdaRCN emerges as the most advantageous solution, achieving the highest PSNR of 38.23 dB while maintaining a significantly low latency of approximately 20 ms. Other CNN-based models (like LAPAR-A [7], DiVANet [41], LatticeNet [8], HPUN-L [72], FMEN [73], OSFFNet [74], and SRConvNet-L [26]) also demonstrate high inference efficiency, but the reconstruction quality is relatively poor. Additionally, the inference latency of transformer-based models is significantly higher than those of CNN-based models, e.g., SwinIR [21] and LBNet [76].

**Table 1:** Quantitative results of advanced lightweight SR models. ★ represents transformer models and * denotes that the results are recomputed according to the description of the referred paper. The best results are marked in red and the second ones are in blue for non-transformer methods. Light blue background indicates the best result among all compared methods, and grey background color denotes Transformer-based methods.

Scales	SISR Models	Annual	# Params	# MultiAdds	Set5		Set14		BSD100		Urban100		Manga109	
			[K]	[G]	PSNR	SSIM	PSNR	SSIM	PSNR	SSIM	PSNR	SSIM	PSNR	SSIM
SR × 2	EDSR-baseline [31]	CVPRW2017	1370	316.2	37.91	0.9602	33.53	0.9172	32.15	0.8995	31.99	0.9270	38.40	0.9766
	CARN [10]	ECCV2018	1592	222.8	37.76	0.9590	33.52	0.9166	32.09	0.8978	31.92	0.9256	38.36	0.9765
	LAPAR-A [7]	NeurIPS2020	548	171.0	38.01	0.9605	33.62	0.9183	32.19	0.8999	32.10	0.9283	38.67	0.9772
	TSAN [71]	TCSVT2021	3990	1011.4	**38.22**	**0.9613**	33.84	0.9196	**32.32**	**0.9015**	**32.77**	**0.9345**	—	—
	SMSR [83]	CVPR2021	985	224.1	38.00	0.9601	33.64	0.9179	32.17	0.8990	32.19	0.9284	38.76	0.9771
	DiVANet [41]	PR2022	902	189.0	38.16	0.9612	33.80	0.9195	32.29	0.9012	32.60	0.9325	39.08	0.9775
	LatticeNet [8]	TPAMI2022	756	169.5	38.15	0.9610	33.78	0.9193	32.25	0.9005	32.43	0.9302	—	—
	FMEN [73]	CVPRW2022	748	172.0	38.10	0.9609	33.75	0.9192	32.26	0.9007	32.41	0.9311	38.95	0.9778
	HPUN-L [72]	AAAI2023	714	151.1	38.10	0.9608	33.78	0.9201	32.28	0.9010	32.49	0.9318	39.08	0.9779
	OSFFNet [74]	AAAI2024	516	83.2	38.11	0.9610	33.72	0.9190	32.29	0.9012	32.67	0.9331	39.09	0.9780
	CoMoNet-S [75]	TCSVT2024	722	31.4	38.06	0.9606	**33.84**	**0.9206**	32.23	0.9003	32.45	0.9315	**39.28**	**0.9783**
	CMSN [82]	SPL2024	4931 *	1133.5 *	38.18	0.9612	33.84	0.9195	32.30	0.9014	32.65	0.9329	39.11	0.9780
	SRConvNet-L [26]	IJCV2025	885	160	38.14	0.9610	33.81	0.9199	32.28	0.9010	32.59	0.9321	**39.22**	0.9779
	AdaRCN (Ours)	—	1315	290.0	**38.23**	**0.9615**	**33.91**	**0.9207**	**32.33**	**0.9016**	**32.76**	**0.9342**	39.15	**0.9785**
	★SwinIR-light [21]	CVPRW2021	878	195.6	38.14	0.9610	33.86	0.9206	32.31	0.9012	32.76	0.9338	39.12	0.9783
	★ LBNet [76]	IJCAI2022	731	153.2	38.05	0.9607	33.65	0.9177	32.16	0.8994	32.30	0.9291	38.88	0.9775
	★ NGswin [78]	CVPR2023	998	140.4	38.05	0.9610	33.79	0.9199	32.27	0.9008	32.53	0.9324	38.97	0.9777
	★ CFIN [79]	TMM2023	675	116.9	38.14	0.9610	33.80	0.9199	32.26	0.9006	32.48	0.9311	38.97	0.9773
	★ DIIN [80]	TIM2024	726	92.1	38.06	0.9610	33.73	0.9189	32.20	0.8998	32.37	0.9301	38.95	0.9778
	★ GMN [81]	ESWA2025	1110	259.0	38.16	0.9611	33.88	0.9201	32.30	0.9012	32.55	0.9325	39.17	0.9781
SR × 3	EDSR-baseline [31]	CVPRW2017	1554	160.4	34.28	0.9263	30.24	0.8405	29.06	0.8044	28.00	0.8493	33.37	0.9432
	CARN [10]	ECCV2018	1592	118.8	34.29	0.9255	30.29	0.8407	29.06	0.8034	28.06	0.8493	33.50	0.9440
	LAPAR-A [7]	NeurIPS2020	594	114.0	34.36	0.9267	30.34	0.8421	29.11	0.8054	28.15	0.8523	33.51	0.9441
	TSAN [71]	TCSVT2021	4174	554.3	**34.64**	0.9282	**30.52**	0.8454	29.20	0.8080	28.55	0.8602	—	—
	SMSR [83]	CVPR2021	993	100.5	34.40	0.9270	30.33	0.8412	29.10	0.8050	28.25	0.8536	33.68	0.9445
	DiVANet [41]	PR2022	949	89.0	34.60	0.9285	30.47	0.8447	29.19	0.8073	28.58	0.8603	33.94	0.9468
	LatticeNet [8]	TPAMI2022	765	76.3	34.53	0.9281	30.39	0.8424	29.15	0.8059	28.33	0.8538	—	—
	FMEN [73]	CVPRW2022	757	77.2	34.45	0.9275	30.40	0.8435	29.17	0.8063	28.33	0.8562	33.86	0.9462
	HPUN-L [72]	AAAI2023	723	69.3	34.58	0.9282	30.46	0.8445	29.19	0.8073	28.39	0.8582	33.96	0.9467
	OSFFNet [74]	AAAI2024	524	37.8	34.58	0.9287	30.48	0.8450	29.21	0.8080	28.49	0.8595	34.00	0.9472
	CoMoNet-S [75]	TCSVT2024	874	16.9	34.44	0.9275	30.40	0.8429	29.15	0.8054	28.49	0.8584	34.16	0.9474
	CMSN [82]	SPL2024	4940 *	499.6 *	34.62	**0.9288**	30.50	0.8452	**29.22**	**0.8082**	**28.60**	**0.8612**	34.12	0.9476
	SRConvNet-L [26]	IJCV2025	906	74.0	34.59	0.9288	30.50	**0.8455**	29.22	0.8081	28.56	0.8600	**34.17**	**0.9479**
	AdaRCN (Ours)	—	1361	137.8	**34.67**	**0.9293**	**30.58**	**0.8465**	**29.25**	**0.8085**	**28.68**	**0.8621**	**34.18**	**0.9483**
	★ SwinIR-light [21]	CVPRW2021	886	87.2	34.60	0.9289	30.54	0.8463	29.19	0.8082	28.66	0.8624	33.98	0.9478
	★ LBNet [76]	IJCAI2022	736	68.4	34.47	0.9277	30.38	0.8417	29.13	0.8061	28.42	0.8559	33.82	0.9460
	★ ESRT [77]	CVPRW2022	770	—	34.42	0.9268	30.43	0.8433	29.15	0.8063	28.46	0.8574	33.95	0.9455
	★ NGswin [78]	CVPR2023	1007	66.6	34.52	0.9282	30.53	0.8456	29.19	0.8078	28.52	0.8603	33.89	0.9470
	★ CFIN [79]	TMM2023	681	53.5	34.60	0.9289	30.45	0.8443	29.18	0.8071	28.49	0.8583	33.89	0.9464
	★ DIIN [80]	TIM2024	735	41.8	34.48	0.9280	30.44	0.8436	29.13	0.8062	28.35	0.8551	33.87	0.9461
	★ GMN [81]	ESWA2025	1120	116.4	34.60	0.9291	30.37	0.8454	29.23	0.8091	28.56	0.8609	34.14	0.9482
SR × 4	EDSR-baseline [31]	CVPRW2017	1518	114.2	31.98	0.8927	28.55	0.7805	27.54	0.7348	25.90	0.7809	30.24	0.9053
	CARN [10]	ECCV2018	1592	90.9	32.13	0.8937	28.60	0.7806	27.58	0.7349	26.07	0.7837	30.47	0.9084
	LAPAR-A [7]	NeurIPS2020	659	94.0	32.15	0.8944	28.61	0.7818	27.61	0.7366	26.14	0.7871	30.42	0.9074
	TSAN [71]	TCSVT2021	4137	414.6	32.40	0.8975	28.73	0.7847	27.67	0.7398	26.39	0.7955	—	—
	SMSR [83]	CVPR2021	1006	57.2	32.12	0.8932	28.55	0.7808	27.55	0.7351	26.11	0.7868	30.54	0.9085
	DiVANet [41]	PR2022	939	57.0	32.41	0.8973	28.70	0.7844	27.65	0.7391	26.42	0.7958	30.73	0.9119
	LatticeNet [8]	TPAMI2022	777	43.6	32.30	0.8962	28.68	0.7830	27.62	0.7367	26.25	0.7873	—	—
	FMEN [73]	CVPRW2022	769	44.2	32.24	0.8955	28.70	0.7839	27.63	0.7379	26.28	0.7908	30.70	0.9107
	HPUN-L [72]	AAAI2023	734	39.7	32.38	0.8969	28.72	0.7847	27.66	0.7393	26.36	0.7947	30.83	0.9124
	OSFFNet [74]	AAAI2024	537	22.0	32.39	0.8976	28.75	0.7852	27.66	0.7393	26.36	0.7950	30.84	0.9125
	CoMoNet-S [75]	TCSVT2024	760	9.7	32.24	0.8952	28.68	0.7827	27.61	0.7350	26.41	0.7945	30.99	0.9138
	CMSN [82]	SPL2024	4952 *	280.5 *	32.41	0.8975	28.77	0.7851	27.68	0.7398	26.44	0.7964	**31.00**	0.9133
	SRConvNet-L [26]	IJCV2025	902	45	**32.44**	**0.8976**	**28.77**	**0.7857**	**27.69**	**0.7402**	**26.47**	**0.7970**	30.96	**0.9139**
	AdaRCN (Ours)	—	1352	77.4	**32.46**	**0.8977**	**28.82**	**0.7863**	**27.74**	**0.7409**	**26.49**	**0.7982**	**31.12**	**0.9156**
	★ SwinIR-light [21]	CVPRW2021	897	49.6	32.44	0.8976	28.77	0.7858	27.68	0.7406	26.47	0.7980	30.92	0.9151
	★ LBNet [76]	IJCAI2022	742	38.9	32.29	0.8960	28.68	0.7832	27.62	0.7382	26.27	0.7906	30.76	0.9111
	★ ESRT [77]	CVPRW2022	751	—	32.19	0.8947	28.69	0.7833	27.69	0.7379	26.39	0.7962	30.75	0.9100
	★ NGswin [78]	CVPR2023	1019	36.4	32.33	0.8963	28.78	0.7859	27.66	0.7396	26.45	0.7963	30.80	0.9128
	★ CFIN [79]	TMM2023	699	31.2	32.49	0.8985	28.74	0.7849	27.68	0.7396	26.39	0.7946	30.73	0.9124
	★ DIIN [80]	TIM2024	747	24.2	32.35	0.8963	28.73	0.7842	27.63	0.7378	26.35	0.7920	30.81	0.9119
	★ GMN [81]	ESWA2025	1134	65.0	32.40	0.8979	28.66	0.7868	27.70	0.7416	26.40	0.7963	30.98	0.9143

### 4.4. Model Analysis

**Figure 8:**
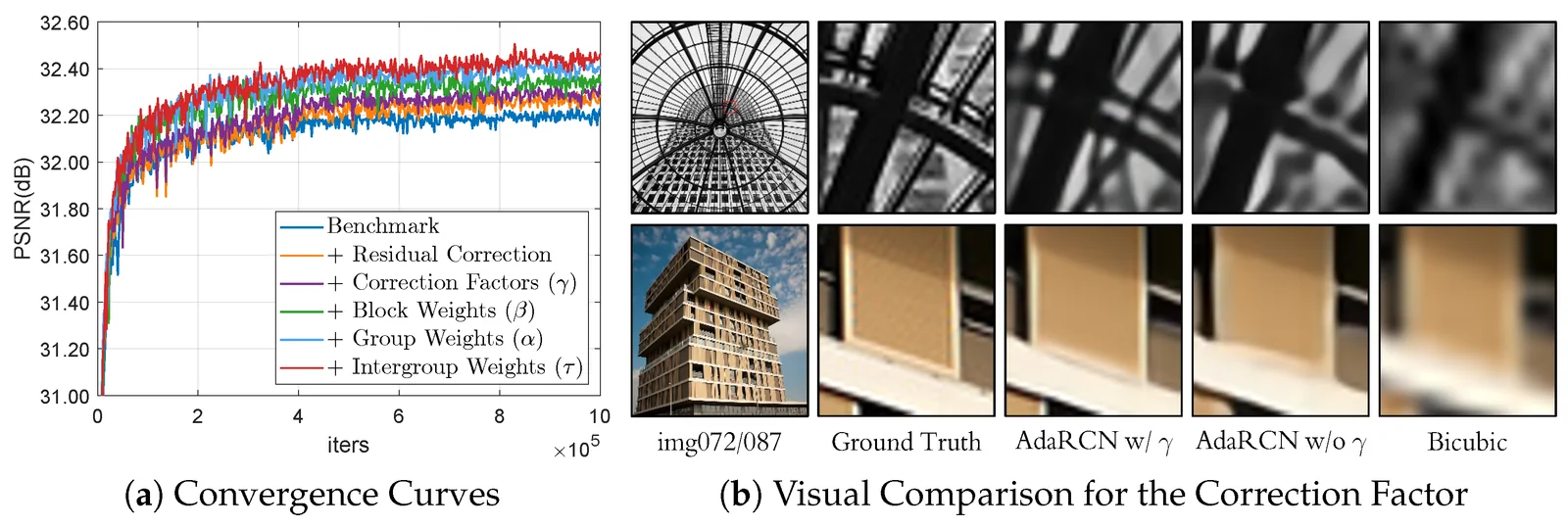
Ablation analysis of our AdaRCN. (**a**) The convergence curves on Set5 [64] for SR × 4, which correspond to Table 2; (**b**) visual comparison of the model with and without the correction factor for interlayer connections (two test images from Urban100 [67], SR × 4).

**Ablation Studies:** Table 2 illustrates the results of ablation experiments on the critical components of the proposed method, including residual correction and various weights for adaptive feature fusion. The results are collected on Set5 [64] with SR × 4. The comparison of residual correction is simply removing it from RCBs, and the control of various weights is setting them to be fixed. It can be observed that both residual correction and adaptive feature fusion can significantly enhance model performance, while the former is able to substantially improve model capability. Specifically, the residual correction (Case 1) improves the PSNR from 32.27 dB to 32.35 dB, while the introduction of the correction factor γ (Case 2) further elevates it to 32.37 dB. Additionally, the various weights (α, β and τ) also contribute positively to the SR accuracy, with the intergroup weights yielding the most substantial improvement. The integration of these weights leads to an overall performance improvement of approximately 0.1 dB. The convergence curves corresponding to the cases in Table 2 are exhibited in Figure 8a. It can be seen that residual correction can effectively promote model optimization, and the complete AdaRCN achieves the fastest convergence.

Beyond quantitative metrics, we also investigate the visual impact of the correction factor γ for interlayer connections, as shown in Figure 8b on two test images from Urban100 [67]. The variant without the correction factor produces noticeable artifacts and blurred textures, particularly in regions with repetitive structures and fine details. In contrast, the recovered images exhibit significantly sharper edges and more faithful texture restoration when γ is enabled, closely approaching the Ground Truth. This visual comparison supports the quantitative finding that the correction factor effectively rectifies the interlayer feature propagation and preserves structural consistency.

**Figure 9:**
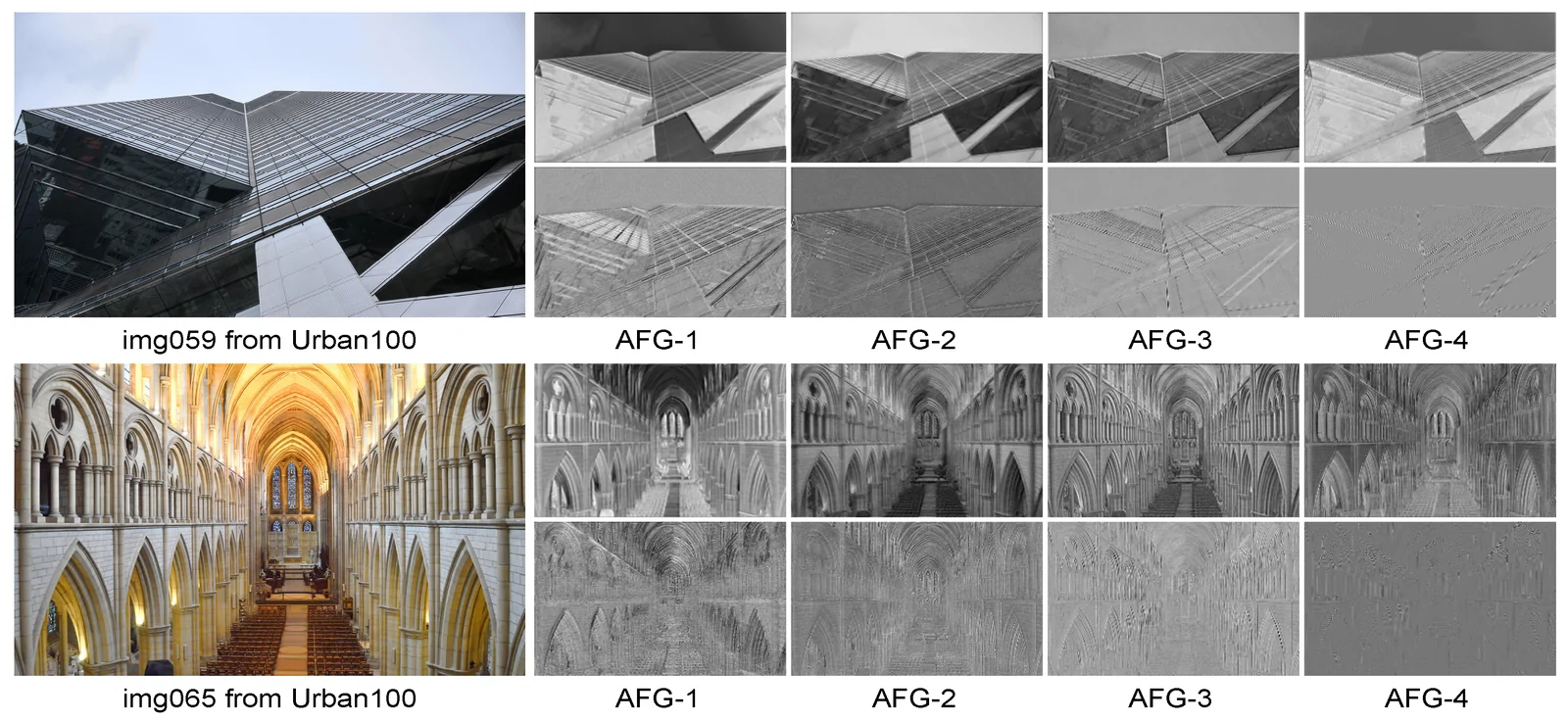
The visualization of residual correction for the output of four AFGs on two test images “img059” and “img065” from Urban100 [67] (SR × 4). For each sample, the first row denotes the input of the AFG, and the second row stands for the output of the *m*-th RCB within this AFG.

**Error Visualization:** Figure 2 illustrates the effect of residual correction within the first and fourth AFGs, where we can observe that the errors of residual features become smaller as the depth of the RCBs increases. Actually, similar phenomena can be observed for different AFGs, as shown in Figure 9. It presents the residuals and errors of the output features of the four AFGs for two test images (“img059” and “img065”) from Urban100 [67], which contain remarkable nonlinear features, such as contours and lines. In general, images containing more nonlinear features are inherently harder to reconstruct with high fidelity, making the errors in the residual mapping intuitively more substantial. However, we can also observe that the intergroup errors gradually diminish as the network depth increases. The underlying reason is that each RCB incrementally corrects the residual bias inherited from the previous mapping and the mapping error is being effectively compensated as the network goes deeper. This observation is consistent with the behavior of error compensation and suggests that the learned correction branch functions as intended. However, we emphasize that these visualizations serve as indirect evidence rather than independent validation of the true residual error, which is not directly measurable. Also, they empirically validate our core assumption that residual errors are non-negligible but can be diminished progressively via our proposed residual correction mechanism.

## 5. Conclusions

In this work, we revisit the commonly used residual learning and feature fusion for efficient SISR tasks. The intuition that there exist heterogeneous errors in residual learning inspires us to explicitly recorrect residual features. To this end, we proposed a lightweight single image super-resolution network termed as AdaRCN, which integrates an adaptive feature fusion mechanism with residual correction and multi-granularity learnable weights. Although the proposed AdaRCN is built entirely upon CNNs without complex structural design, it accomplishes superior performance over advanced transformer-based models with modest overhead. Extensive experiments on benchmark datasets demonstrate that our AdaRCN achieves superior SR performance against representative SR methods with moderate model parameters and competitive computational efficiency. More importantly, benefiting from its concise design, our model offers comparable parallelization efficiency and low-latency performance to standard CNNs. This work demonstrates that the representational capability of traditional CNNs still maintains potential for further exploration, which is favorable for the practical application and deployment of efficient SISR tasks.

## Figures and Tables

**Table 2:** Ablation study of residual correction and various learnable weights for adaptive feature fusion with SR × 4. Please refer to Figure 3 for the structural details of our AdaRCN.

Cases	Benchmark	✓	✓	✓	✓	✓	✓
1	+Residual Correction		✓	✓	✓	✓	✓
2	+Correction Factor (γ)			✓	✓	✓	✓
3	+Block Weights (β)				✓	✓	✓
4	+Group Weights (α)					✓	✓
5	+Intergroup Weights (τ)						✓
Results	□ PSNR (↑) on Set5 [64]	32.27	32.35	32.37	32.40	32.42	32.46
	□ SSIM (↑) on Set5 [64]	0.8954	0.8966	0.8969	0.8971	0.8973	0.8977

## Data Availability

The original contributions presented in this study are included in the article. Further inquiries can be directed to the corresponding author.
